# Stemness-Associated Biomarkers in Breast Cancer Liver Metastasis: Hepatic Adaptation, Prognostic Relevance, and Monitoring

**DOI:** 10.3390/diagnostics16193076

**Published:** 2026-09-22

**Authors:** Bin Hu, Jing Hou, Shihang Hu

**Affiliations:** 1 Graduate School, Guizhou Medical University, Guiyang 550004, China; 2 Department of Breast Surgery, Guizhou Provincial People’s Hospital, Guiyang 550002, China

**Keywords:** breast cancer liver metastasis, cancer stemness, circulating tumor cells, liquid biopsy, hepatic niche

## Abstract

The clinical assessment, treatment, and prognostic evaluation of breast cancer liver metastasis (BCLM) remain clinically challenging. Hepatic involvement is often identified only after substantial intrahepatic tumor burden, radiologically evident parenchymal involvement, abnormal liver function, or symptoms attributable to hepatic impairment have developed. Standard evaluation relies on molecular subtype, tumor burden, liver function, imaging, metastatic-site biopsy, receptor reassessment, and selected genomic testing; however, these variables do not fully explain why only a subset of disseminated tumor cells survives within the hepatic microenvironment, persists after therapy, and contributes to liver-dominant recurrence. This review examines CD44/CD24 phenotypic states, aldehyde dehydrogenase (ALDH) activity and isoform-specific expression, epithelial cell adhesion molecule (EpCAM)-positive and heterogeneous circulating tumor cells, claudin-2-associated hepatic tropism, and cellular interactions within the liver metastatic niche. We discuss how stemness-associated phenotypes may aid in interpreting delayed detectability, residual-cell persistence, treatment tolerance, and hepatic recurrence, while emphasizing that no single marker is sufficient for clinical stratification. Tissue reassessment, liquid biopsy, imaging, and clinicopathological information should be interpreted as complementary layers. Further progress requires BCLM-specific prospective cohorts, standardized tissue- and blood-based assays, paired primary and metastatic samples, serial liquid biopsy, and liver-specific endpoints that link residual disease biology to clinically meaningful monitoring and management.

## 1. Introduction

Breast cancer remains one of the most common malignancies among women worldwide, and distant metastasis is the principal determinant of breast cancer-related death [1]. The liver is a clinically important visceral site because hepatic involvement can rapidly affect systemic therapy tolerance, local treatment feasibility, and survival [2,3]. Breast cancer liver metastasis (BCLM) thus represents not merely an anatomic pattern of dissemination but a disease state in which tumor burden, molecular subtype, hepatic function, residual disease, and treatment resistance are tightly intertwined.

Clinical management incorporates receptor reassessment and HER2-low interpretation [4,5,6], selected ESR1 or PIK3CA testing when clinically relevant [4,7,8], together with organ function, symptoms, prior treatment, and disease tempo. These factors guide treatment, but they do not explain why only some disseminated tumor cells survive in hepatic sinusoids, interact with local cells, persist after therapy, and drive liver-predominant progression. Established biomarkers such as ER, PR, HER2, ESR1, and PIK3CA are routinely used to characterize metastatic breast cancer and guide treatment, whereas CD44/CD24, ALDH-related measurements, EpCAM/CTCs, and CLDN2 remain mainly investigational markers.

This review discusses BCLM from integrated perspectives encompassing clinical management, cancer stemness, circulating tumor cell (CTC) biology, liquid biopsy, liver tropism, and hepatic niche adaptation. CD44/CD24, ALDH-related measurements, EpCAM-positive or heterogeneous CTCs, and claudin-2 (CLDN2) are discussed as complementary biological layers rather than interchangeable diagnostic markers. We aim to link clinical behavior to underlying molecular and cellular processes. Such links may guide future BCLM-specific risk stratification, monitoring, and validation.

## 2. Clinical Context and Biological Heterogeneity of Breast Cancer Liver Metastasis

### 2.1. Clinical Burden, Delayed Detectability, and Treatment Constraints

BCLM presents a clinical challenge because hepatic progression may remain clinically silent until lesions become radiographically or biochemically evident. At that stage, patients may already exhibit pain, biliary obstruction, cholestasis, impaired synthetic function, pseudocirrhosis-like changes, or hepatic dysfunction. These complications narrow systemic and local treatment options. Compared with bone-only progression, liver-dominant disease often narrows the therapeutic window for endocrine therapy, chemotherapy, HER2-targeted agents, antibody-drug conjugates, immunotherapy, and liver-directed interventions.

Liver-directed treatments illustrate the potential—and limitations—of lesion-directed management. Bale et al. reviewed studies published between 2000 and June 2019 and included 44 clinical studies from 201 screened records: 24 on hepatic resection, 9 on radiofrequency ablation, 1 on cryoablation, 5 on radioembolization or selective internal radiation therapy (SIRT), 2 on transarterial chemoembolization (TACE), 2 on brachytherapy, and 4 on stereotactic body radiotherapy (SBRT) [8]. Within the surgical evidence summarized in that review, a systematic review of 956 patients who underwent resection reported a median overall survival (OS) of 35.1 months, a median disease-free survival (DFS) of 21.5 months, and median 1-, 2-, 3-, and 5-year OS rates of 84.6%, 71.4%, 52.9%, and 33%, respectively [8]. A European case-matched study reported longer survival with systemic therapy plus hepatic resection than with systemic therapy alone, with a median OS of 82 vs. 31 months and 5-year OS rates of 69% vs. 24% [9].

The case-control study by Sadot et al. illustrates why these results require cautious interpretation. Among 2150 patients with BCLM, only 167 (approximately 8%) had isolated liver metastasis; 69 underwent resection and/or ablation, and 98 received medical therapy [9]. Although the liver-directed treatment group achieved a median recurrence-free interval of 28.5 months and approximately 15% remained recurrence-free at 5 years, OS was not clearly improved compared with medical therapy alone: median OS was 50 vs. 45 months, and 5-year OS rates were 38% vs. 39% [10]. A reconstructed individual patient data meta-analysis of four propensity score-matched studies reported improved OS in selected surgical patients, including 205 surgical and 291 non-surgical cases, with an OS hazard ratio of 0.40 (95% CI, 0.32–0.51) favoring surgery [11]. These findings support a selective role for liver-directed therapy while also underscoring the importance of patient selection, tumor biology, extrahepatic disease, hepatic reserve, response to systemic therapy, and the risk of residual disease.

Recent data further support this interpretation. Schullian et al. treated 42 consecutive female patients with 110 BCLM lesions using stereotactic radiofrequency ablation (SRFA), achieving a technical success rate of 100%, initial ablation in 107 of 110 lesions, local recurrence in 8 of 110 tumors (7.3%), and 1-, 3-, and 5-year OS rates of 84.1%, 49.3%, and 20.8%, respectively, with a median OS of 32.3 months [12]. For SIRT, a meta-analysis of 24 studies from 14 institutions involving 412 patients reported a median post-treatment survival of 9.8 months; outcomes were poorer in patients with extrahepatic disease than in those with liver-only disease (5.3 vs. 15.0 months) and in those with a liver tumor burden greater than 25% than in those with a burden of 25% or less (6.8 vs. 10.5 months) [13]. Thus, local or regional therapies can control visible lesions in selected patients, but their clinical benefit remains dependent on disease burden and systemic tumor biology.

### 2.2. Clinical Detection and Monitoring of BCLM

The clinical assessment of BCLM usually starts with imaging and routine laboratory tests. Contrast-enhanced computed tomography, liver magnetic resonance imaging, and selected positron emission tomography/computed tomography are used to define the number, size, and distribution of liver lesions. Liver enzyme levels, bilirubin, tumor markers, and response assessment help clinicians judge disease burden, liver function, and treatment tolerance. Metastatic-site biopsy also allows for reassessment of ER, PR, HER2, and HER2-low status in metastatic lesions [4,5,6,14], while selected molecular testing may further inform treatment when clinically indicated [4,7,8].

Each method answers a different clinical question. Imaging shows macroscopic disease features, including lesion growth, mixed response, pseudocirrhosis, biliary obstruction, and recurrence after local therapy. It cannot show whether residual cells have gained stem-like or treatment-tolerant features. Biopsy remains important when receptor conversion or genomic profiling may change treatment, but repeated liver sampling is not always practical. Lesion location, bleeding risk, impaired liver function, and the patient’s general condition may limit its use. In addition, a single biopsy only samples part of a lesion at one time and may miss spatial heterogeneity or later phenotypic changes.

Liquid biopsy can partly fill this gap. CTC analysis retains cellular information and may reveal epithelial, mesenchymal, stemness-associated, clustered, or immune-evasive features [14,15]. ctDNA is more useful for tracking tumor burden, clonal evolution, actionable mutations, and selected resistance mechanisms [14]. In BCLM, these approaches should be viewed as complementary. Tissue biopsy defines lesion biology and receptor status; CTCs describe cells with dissemination potential; ctDNA reflects molecular burden and evolution; and imaging remains essential for evaluating visible liver disease.

### 2.3. Molecular Subtypes and Biomarker Interpretation

BCLM progression differs markedly across the main breast cancer subtypes. HR-positive disease may remain controlled for years, but endocrine resistance can later shift the clinical course toward liver-dominant progression. HER2-positive disease has changed substantially with HER2-targeted therapy and antibody-drug conjugates, although receptor discordance, visceral progression, and acquired resistance remain common clinical concerns. TNBC is more often associated with early visceral spread, immune and stromal remodeling, and fewer durable targeted options. We therefore interpret stemness-associated markers as dynamic clues shaped by subtype, treatment pressure, and metastatic site, rather than as fixed clinical labels.

CD44/CD24 is a classical breast cancer stem-like surface phenotype. Its tumor-initiating properties were established in foundational studies [16]. Subsequent studies linked CD44/CD24-associated states to plasticity and treatment resistance [17,18], while BCLM-related preclinical work associated CD44high/CD24− states with invasion and hepatic metastasis [19,20]. ALDH is similarly assay-dependent: ALDEFLUOR reports enzymatic activity, whereas ALDH1A1 and ALDH1A3 expression represent isoform-specific readouts and should not be interpreted interchangeably. Claudin-2 differs in that it links tumor cell state to liver-proximal organotropism. Tabaries et al. experimentally demonstrated an association between Claudin-2 and breast cancer cell-hepatocyte interactions, and Kimbung et al. linked Claudin-2 expression to early hepatic recurrence in patient cohorts [21,22].

These markers should not be regarded as interchangeable clinical biomarkers. CD44/CD24 and ALDH-related measurements primarily support the interpretation of plasticity and treatment tolerance; EpCAM/CTCs provide a liquid biopsy-based window into dissemination and epithelial detectability; and Claudin-2 is more appropriately considered an organotropic indicator related to hepatocyte interaction and hepatic colonization.

## 3. Stemness-Related Markers in BCLM

### 3.1. Stemness Plasticity During Metastatic-State Transition

Stem-like traits include self-renewal, phenotypic plasticity, EMT, and resistance to apoptosis [16,17,18,]. Treatment-tolerant states may additionally involve autophagy [23], metabolic remodeling [24,25], JAK-STAT signaling [26], and immune-evasive programs [27]. These traits are relevant to BCLM but do not operate in isolation. Disseminated cells must resist anoikis and shear stress, interact with vascular and immune components, arrest within the hepatic microvasculature, and adapt to a niche that may promote clearance, dormancy, or outgrowth.

Metastatic-state transition refers to the remodeling of tumor-cell phenotypes from invasive cells in the primary lesion to single CTCs or CTC clusters, sinusoid-arrested disseminated cells, micrometastatic cells, dormant or residual cells, and clinically detectable metastases. These markers provide different types of information rather than a single biomarker signal. CD44/CD24 mainly reflects invasion, EMT-related plasticity, and treatment tolerance. ALDH-related features reflect aldehyde metabolism and stress adaptation, but their interpretation is assay-dependent. EpCAM-positive CTCs help identify circulating tumor cells but may underrepresent EpCAM-low or mesenchymal populations. Claudin-2 is more liver-oriented because it is associated with hepatocyte interaction and hepatic colonization. We therefore interpret these markers according to sample source and timing, including primary tumors, liver metastasis biopsies, CTCs, ctDNA, extracellular vesicles, and post-treatment residual samples. Figure 1 summarizes a conceptual framework linking stemness-associated cellular states, CTC heterogeneity, hepatic sinusoidal arrest, and niche-driven dormancy or outgrowth during the transition from primary breast tumors to clinically detectable BCLM.

This schematic illustrates a proposed biological framework linking stemness-associated phenotypic states with hepatic niche adaptation during breast cancer liver metastasis (BCLM). Dissemination begins with primary tumor invasion enriched for plastic and stemness-associated features, including CD44^high/CD24^low phenotypes, ALDH enzymatic activity, and epithelial–mesenchymal transition (EMT)-related traits. Circulating tumor cells (CTCs) represent heterogeneous metastatic intermediates, including epithelial CTCs detectable by epithelial markers, hybrid EMT-shifted populations, and stem-like CTCs or CTC clusters with enhanced metastatic potential. Following hepatic sinusoidal arrest, disseminated cells interact with hepatocytes, sinusoidal endothelial cells, Kupffer cells, hepatic stellate cells, and other niche components, where adhesion-related mechanisms, extracellular matrix remodeling, chemokine signaling, and inflammatory cytokine pathways may contribute to dormancy, survival, or metastatic outgrowth. These processes may ultimately contribute to clinically detectable liver metastasis, residual disease persistence, treatment resistance, and liver-dominant recurrence. As a conceptual framework, the figure integrates BCLM-specific evidence with broader breast cancer and metastatic-niche mechanisms and does not imply equivalent BCLM-specific validation for every mechanism shown.

### 3.2. CD44/CD24 States in Metastatic Spread

In breast cancer studies, CD44 and CD24 are often used to identify cell states associated with invasion, EMT, and treatment tolerance. CD44 is involved in adhesion, migration, survival, and cell-matrix interactions, whereas low or absent CD24 expression is frequently combined with CD44 positivity to enrich for breast cancer stem-like populations [16,17]. In the seminal study by Al-Hajj et al., CD44+/CD24−/low/Lineage- cells were prospectively identified in 8 of 9 patient samples; as few as 100 cells with this phenotype formed tumors in immunodeficient mice, whereas tens of thousands of cells with alternative phenotypes did not [16].

In BCLM-related preclinical studies, transforming growth factor beta 1 (TGF-beta1) increased the CD44high/CD24− subpopulation; upregulated stemness-associated transcripts, including NANOG, POU5F1/OCT4, and SOX2; and promoted local invasion and liver metastasis in breast cancer models [19]. CD44-high cells were enriched at the invasive front of primary tumors, and clusters of CD44-positive breast cancer cells were observed in mouse livers [18]. Extracellular vesicles derived from CD44+/CD24− breast cancer stem cells can also deliver miR-197, suppress PPARG, promote EMT, and enhance hepatic metastasis in preclinical models [20].

CD44+/CD24−/low cells are present in several breast cancer subtypes, although they usually account for only a small fraction of the tumor. In a prospective flow cytometry study of 143 treatment-naive tumors, this population was most frequent in luminal B tumors (3.4%), followed by TNBC (1.7%), HER2-enriched tumors (1.6%), and luminal A tumors (1.3%) [28]. This finding is important because small stem-like populations may be missed in bulk tumor analyses. In BCLM, CD44/CD24 is better viewed as a clue to invasive plasticity, adhesion, EMT-related change, and residual disease biology, not as a stand-alone marker for risk stratification or liver-directed treatment selection.

### 3.3. ALDH Activity, Isoform Expression, and Treatment Tolerance

ALDH has long been used as a functional marker of stem-like cells in breast cancer, but its interpretation depends on how it is measured. The ALDEFLUOR assay detects cellular aldehyde dehydrogenase activity rather than a specific ALDH isoform. Ginestier et al. showed that ALDEFLUOR-positive breast cancer cells are enriched for self-renewing and tumor-initiating properties [29]. Subsequent studies, however, showed that this activity should not be equated with ALDH1A1 expression.

Marcato et al. found that ALDH1A3 expression correlated more closely with ALDEFLUOR activity than ALDH1A1, and that ALDH1A3 knockdown reduced ALDEFLUOR activity in breast cancer cell lines [30]. Zhou et al. further showed that several ALDH isoforms can contribute to the ALDEFLUOR signal and that their relative contribution varies across cancer types [31]. This distinction also matters clinically: ALDH1A1 protein and transcript expression have shown different associations with outcome in breast cancer cohorts [32]. Together, these findings support treating ALDH enzymatic activity and isoform-specific expression as related but non-equivalent readouts.

For BCLM, the available evidence remains indirect. ALDH activity and isoform-specific expression may reflect stem-like, stress-adaptive, or treatment-tolerant states, but neither has a validated liver-specific threshold or established clinical role. Future studies should therefore report the assay and isoform used, because measurements obtained from different analytical approaches may represent distinct biological features.

### 3.4. EpCAM, CTC Detection, and Epithelial Plasticity

EpCAM is relevant to the interpretation of BCLM for both biological and technical reasons. It is a cell-surface adhesion molecule expressed by many epithelial tumors and is included in some breast cancer stem cell marker panels [17]. It is also a core capture marker in widely used CTC enrichment platforms [15,33]. This dual role makes EpCAM useful but potentially misleading: EpCAM positivity facilitates detection of epithelial tumor cells in circulation, yet it does not capture the full spectrum of dissemination-competent phenotypes.

During EMT or partial EMT, tumor cells may downregulate epithelial markers, including EpCAM, while acquiring mesenchymal, invasive, or immune-evasive phenotypes [33,34,35,36]. EpCAM-dependent platforms may therefore preferentially isolate CTCs with pronounced epithelial characteristics and underrepresent EpCAM-low, mesenchymal-like, or hybrid epithelial–mesenchymal cells. This is particularly relevant to BCLM, as cells capable of withstanding shear stress, evading anoikis, and adapting to the hepatic sinusoids may not be those most efficiently captured by epithelial marker-based methods.

CTC rarity further complicates interpretation. In metastatic breast cancer, the CellSearch threshold of at least 5 CTCs per 7.5 mL of blood has been widely used for prognostic stratification [15]. CTC clusters add further complexity: although they may account for only a small proportion of CTC events, approximately 3%, they exhibit disproportionately greater metastatic potential than single CTCs [35]. Accordingly, EpCAM should be regarded as a platform-dependent marker of epithelial detectability and a partial entry point into CTC biology. A negative EpCAM-dependent result should not be interpreted as indicating the absence of dissemination risk.

### 3.5. Multi-Marker Interpretation Beyond Single Markers

These markers reflect different aspects of BCLM biology and should therefore be interpreted together rather than individually. Tissue staining can show where marker-positive cells are located in primary tumors or liver metastases. CTC analysis adds information on disseminated-cell phenotypes and can be repeated during follow-up. ctDNA is useful for tracking molecular burden, clonal change, and resistance-related mutations, although it does not directly measure stemness. Imaging remains necessary for assessing lesion growth, distribution, and treatment response. These findings still need to be read together with subtype, liver function, previous treatment, disease-free interval, and extrahepatic disease. However, this multi-marker framework remains some distance from routine clinical practice. Existing studies provide mechanistic and independent-cohort evidence for selected markers such as CLDN2 [21,22], while some BCLM prognostic studies illustrate strategies for internal and external validation [37,38]. Nevertheless, the stemness-associated and circulating biomarkers reviewed here remain at different stages of translation and have not been validated as a stand-alone clinical decision tool. We therefore propose a staged translational pathway. Candidate biomarkers should first be identified through molecular studies for their potential BCLM-related prognostic or monitoring relevance, with the assay, sample source, disease stage, and liver-specific endpoints clearly defined. They should then undergo internal validation in well-defined BCLM cohorts using standardized measurements and be assessed for added value beyond established clinicopathological variables. Reproducibility and generalizability should subsequently be tested in independent, preferably multicenter cohorts and across different testing settings. Only after prospective validation and demonstration of clinical utility should these biomarkers be considered for clinical integration, where they would complement, rather than replace, imaging, metastatic-site pathology, receptor reassessment, and actionable genomic testing. The characteristics, assay approaches, interpretations, and limitations of these biomarkers are summarized in Table 1.

### 3.6. Other Stemness Pathways and Treatment Resistance

Other stemness-associated pathways provide mechanistic context for treatment resistance and metastatic behavior. Core regulators such as NANOG, OCT4/POU5F1, and SOX2, together with Notch, Wnt/beta-catenin, and Hedgehog signaling, contribute to breast cancer stem-cell plasticity and self-renewal [18]. Additional studies have linked autophagy to breast cancer progression and treatment response [23], MYC/TET3-regulated oxidative phosphorylation to TNBC stemness and cisplatin resistance [24], mitochondrial lactate oxidation to stemness potential in metastatic breast cancer [25], JAK-STAT signaling to chemotherapy-resistant states in inflammatory breast cancer [26], and CD44/PD-L1-mediated networks to drug resistance and immune evasion in breast cancer stem cells [27]. These findings are mechanistically informative but have not been validated as BCLM-specific biomarkers.

Together, these studies provide a broader biological context for residual disease and recurrence, in which cellular plasticity, metabolic adaptation, immune escape, and treatment tolerance may coexist. However, direct evidence linking these pathways to post-treatment recurrence specifically in BCLM remains limited. Prospective BCLM studies should therefore evaluate candidate stemness and residual-disease features using clearly defined assays and liver-specific clinical endpoints, rather than assuming that findings from general breast cancer models directly predict hepatic recurrence.

## 4. Hepatic Niche Adaptation and Organ-Tropic Molecular Clues

### 4.1. Claudin-2-Associated Liver Tropism and Hepatic Colonization

Tumor cell plasticity and therapeutic tolerance do not fully account for why disseminated cells preferentially persist in the liver. Claudin-2 provides a mechanistic link between tumor cell state and organ-specific adhesion. Tabaries et al. demonstrated that Claudin-2 enhances interactions between breast cancer cells and hepatocytes, thereby facilitating liver metastasis [21]. Claudin-2 expression was increased in liver metastases, but not in skin metastases, relative to matched primary tumors, and was preferentially expressed in liver-tropic breast cancer cell populations. Partial reduction of Claudin-2 in primary hepatocytes by approximately 25–50% attenuated the adhesion of liver-aggressive breast cancer cells, supporting a heterotypic tumor cell-hepatocyte interaction model [21].

Kimbung et al. provided clinical evidence by analyzing 304 women with metastatic breast cancer and 237 premenopausal women with early-stage node-negative breast cancer. CLDN2 expression was significantly upregulated in liver metastases compared with other metastatic sites, and high expression was associated with shorter metastasis-free intervals and shorter liver-specific recurrence intervals [22]. Afadin cooperates with Claudin-2 to promote breast cancer metastasis [39], while the circROBO1/KLF5/FUS feedback loop regulates BCLM in part through Afadin-related mechanisms [40]. Taken together, these findings suggest that CLDN2 is more than a marker that becomes upregulated only after liver metastasis has occurred. Experimental gain- and loss-of-function studies show that CLDN2 facilitates breast cancer cell adhesion to hepatocytes and early survival after hepatic seeding, while disruption of CLDN2 or its functional domains reduces liver metastatic capacity in vivo [21,39]. More recently, a multi-organ experimental model showed that CLDN2-overexpressing MDA-MB-231 cells preferentially localized to the liver compartment compared with bone and lung compartments, providing independent functional support for a role of CLDN2 in breast cancer organotropism [41]. The association between CLDN2 expression in primary breast tumors and subsequent liver-specific recurrence further suggests that this phenotype can precede clinically apparent liver metastasis [22]. Nevertheless, increased CLDN2 expression in established liver metastases may reflect both selection of pre-existing CLDN2-positive populations and further adaptation within the hepatic microenvironment. CLDN2 is therefore not only functioning as a mediator of hepatic colonization, but also acting as a liver-tropic molecular clue.

### 4.2. Hepatocyte-, Stromal-, Immune-, and Metabolic Cues in Hepatic Adaptation

The hepatic metastatic niche comprises malignant cells, hepatocytes, liver sinusoidal endothelial cells, Kupffer cells, recruited macrophages, neutrophils, fibroblasts, hepatic stellate cells, extracellular matrix components, soluble mediators, and metabolic cues. These elements influence whether disseminated cells are cleared, transiently retained, enter dormancy, or proliferate. Hepatocytes can support liver colonization through adhesion-related and metabolic interactions, with Claudin-2 representing one example of hepatocyte–tumor cell contact [21]. Kupffer cells and recruited macrophages may participate in early antitumor surveillance, whereas tumor-derived signals can subsequently reprogram hepatic macrophages toward tumor-supportive states that promote tumor-cell survival and metastatic niche formation [42].

These findings suggest that BCLM should not be explained by tumor-cell markers alone. Xuan et al. identified SCGB3A1-Epi and KLK10-Epi as epithelial subpopulations associated with liver metastasis in breast cancer and pancreatic ductal adenocarcinoma, respectively, with their reported crosstalk with fibroblasts computationally inferred from single-cell RNA-sequencing ligand–receptor analysis [43]. Although this study did not directly test stemness markers, the breast cancer findings support a niche-centered view of BCLM. We read these findings together with hepatic stress signals, including nutrient competition, oxidative stress, lipid-rich conditions, and treatment injury. Such conditions may favor tumor cells with greater metabolic and redox adaptability.

The metabolic state of the liver may also influence which tumor cells survive after dissemination. In fatty liver or metabolic dysfunction-associated steatotic liver disease (MASLD), hepatocytes contain abundant lipids, and these lipids can become a fuel source for metastatic cells. Li et al. showed that steatotic hepatocytes transfer lipids to breast cancer cells and promote liver metastatic growth [44]. Breast cancer studies have also linked oxidative phosphorylation to therapy-resistant states and mitochondrial lactate oxidation to stemness potential [24,25]. These findings do not establish hepatic steatosis as a validated biomarker for BCLM, but they support the possibility that a lipid-rich hepatic environment may favor metabolically adaptable tumor-cell states.

### 4.3. Immune and Stromal Niches in BCLM

The hepatic immune environment can influence whether disseminated tumor cells are cleared or supported during metastatic colonization. Kupffer cells and recruited monocyte-derived macrophages may participate in early antitumor surveillance, but tumor-derived signals can also reprogram these cells toward tumor-supportive states. Hepatic macrophages can thereby contribute to tumor-cell survival, immune suppression, and metastatic niche formation [42].

These observations suggest a possible reciprocal interaction between tumor-cell states and the hepatic immune niche. Tumor-derived signals can recruit or reprogram hepatic macrophages [42], whereas macrophage-derived mediators may support tumor-cell survival and an immunosuppressive metastatic environment [42,43]. TGF-β signaling has been associated with expansion of CD44high/CD24− breast cancer cell populations [19]. IL-10-related pathways have also been implicated in immunosuppressive liver metastatic microenvironments [45]. CD44/PD-L1-mediated networks have also been implicated in immune evasion of breast cancer stem cells [27]. However, direct evidence for a stemness-specific macrophage feedback loop in BCLM remains limited, and this framework should therefore be regarded as mechanistic rather than clinically validated.

Stromal and vascular conditions also influence hepatic colonization by shaping tumor-cell arrest, adhesion, and survival within the liver. Among the adhesion-related mechanisms discussed in this review, Claudin-2 has direct experimental support in BCLM, where it facilitates breast cancer cell interactions with hepatocytes [21]. Extracellular vesicles and tumor-derived secreted factors may further modify metastatic microenvironments [20,46]. For example, breast cancer stem cell-derived vesicles carrying miR-197 promote EMT and liver metastasis by targeting PPARG [20]. Additional studies of the breast cancer secretome also show that tumor-derived factors can affect fibroblasts, immune cells, endothelial cells, and other stromal components [46]. We therefore interpret stemness-associated markers together with immune, stromal, and vascular signals, rather than as isolated markers.

## 5. Clinical Implications for Risk Interpretation, Monitoring, and Therapeutic Management

### 5.1. Tissue and Liquid Biopsy in Clinical Assessment

Tissue biopsy and liquid biopsy provide different types of information in BCLM. Liver biopsy remains important when metastatic disease needs pathological confirmation or when reassessment of ER, PR, HER2, receptor heterogeneity, or selected molecular alterations may affect treatment [4,5,6,7,8,47]. It gives direct information from a metastatic lesion. However, it is invasive and difficult to repeat. In liver metastases, sampling may also be limited by lesion location, liver function, bleeding risk, and the patient’s general condition.

Liquid biopsy is useful because it can be repeated during follow-up. CTCs, ctDNA, circulating RNA, extracellular vesicles, and other blood-based analytes can reflect dissemination, tumor burden, clonal change, resistance, and treatment response [14,48,49,50,51]. CTCs retain cellular information and can be assessed for EpCAM, EMT, stemness-associated markers, immune-related features, and clusters. ctDNA is better suited for tracking somatic mutations, copy-number changes, methylation, fragment patterns, and clonal burden. Extracellular vesicles and circulating RNA may add information on cell communication and transcriptional activity, although these assays are less standardized. In practice, we interpret these tests side by side. Tissue shows lesion status, CTCs describe disseminating cells, ctDNA follows molecular evolution, and imaging shows visible disease behavior.

### 5.2. CTCs and ctDNA During Follow-Up

CTCs offer a cellular view of metastatic spread. Unlike ctDNA, they preserve cell morphology, protein expression, RNA profiles, and cluster structure. This makes it possible to study epithelial features, partial EMT, stemness-associated markers, immune interactions, and multicellular clusters [15,33,34,35,36]. Their use is still limited by low abundance and platform bias. Different enrichment methods capture different CTC populations, and EpCAM-based systems may miss EMT-shifted cells. Heterotypic clusters with neutrophils, platelets, or stromal cells are also difficult to capture reproducibly. For BCLM, we do not regard CTCs as a stand-alone test for early detection. Their stronger value may lie in follow-up. Persistent CTC clusters or stemness-enriched CTC phenotypes in patients with stable imaging may identify candidate biological features for prospective evaluation in longitudinal BCLM studies, but they should not currently determine surveillance intensity.

ctDNA provides a different type of information. It can track tumor burden, clonal evolution, genomic alterations, and resistance-related changes with a minimally invasive test [48,49,50,51]. In HR-positive/HER2-negative metastatic disease, ESR1 testing can guide endocrine therapy selection, and PIK3CA testing can support PI3K pathway-targeted treatment decisions [7,52]. However, ctDNA does not show whether tumor cells have stem-like, EMT-shifted, or hepatic niche-adapted features. Its interpretation in BCLM also needs caution. Liver injury, biliary obstruction, inflammation, treatment-related liver damage, and clonal hematopoiesis may all affect the cfDNA background or introduce non-tumor variants if matched leukocyte filtering is not used [48,49,50,51].

The timing of blood sampling may influence the interpretation of liquid biopsy results after liver-directed therapy [48,49,50,51]. Ablation, stereotactic body radiotherapy (SBRT), and other local treatments can induce tumor cell death, hepatic injury, and inflammatory responses, potentially altering circulating DNA measurements during the early post-treatment period. In patients with hepatocellular carcinoma, Nakatsuka et al. found that plasma cell-free DNA (cfDNA) levels increased significantly one day after radiofrequency ablation (RFA) or transarterial chemoembolization (TACE), from a median of 39.2 to 96.2 ng/mL after RFA and from 48.7 to 248.7 ng/mL after TACE (both *p* < 0.001) [53], indicating substantial acute treatment-related changes in circulating DNA. In a prospective translational study of 35 patients with colorectal cancer undergoing local treatment of liver and/or lung metastases, including resection, RFA, and SBRT, Boysen et al. reported that detectable post-treatment ctDNA was associated with a shorter time to recurrence and remained independently associated with a higher risk of recurrence in multivariable analysis [54]. Although these findings were derived from hepatocellular carcinoma and colorectal cancer metastases rather than BCLM, they suggest that liquid biopsy measurements obtained soon after local therapy should be interpreted cautiously in relation to treatment timing and clinical context. The optimal timing of post-treatment liquid biopsy in BCLM requires prospective BCLM-specific kinetic studies.

### 5.3. Clinical Risk Assessment in BCLM

Imaging remains the main tool for assessing visible liver disease. CT, MRI, and selected PET/CT help define tumor number, distribution, treatment response, progression pattern, liver-dominant disease, mixed response, pseudocirrhosis, biliary obstruction, and recurrence after local treatment. MRI-based radiomics and machine-learning approaches have been investigated for recurrence-risk estimation [55], while digital pathology models have been developed for metastatic-relapse prediction [56]. In BCLM, we view these methods as supportive tools. They should add to, rather than replace, clinical and biological interpretation.

Population-based BCLM studies show why risk interpretation should not rely on a single variable. Ji et al. analyzed 2398 patients with de novo BCLM from the SEER database and developed a prognostic score. The low-, intermediate-, and high-risk groups had median OS values of 44, 18, and 7 months, with 3-year OS rates of 56%, 23%, and 7%, respectively [37]. Chen et al. used 4817 SEER cases and 124 external validation cases from two Chinese hospitals to build random forest models. The models reached internal test AUCs of 0.763–0.787 and external validation AUCs of 0.779–0.815. Chemotherapy, age, subtype, and surgery were the main contributors [38]. These studies illustrate how BCLM prognostic models can progress from model development and internal testing to independent external validation before clinical implementation. Importantly, the variables included in these models were predominantly clinicopathological and treatment-related, and they did not validate the stemness-associated or circulating biomarkers discussed in this review. Accordingly, these biomarkers should currently be regarded as candidates for further translational evaluation rather than components of an established clinical risk model. Figure 2 therefore presents a descriptive multimodal framework that distinguishes established clinical assessment from investigational biological information and emphasizes their complementary, non-equivalent roles in interpreting visible disease burden, metastatic lesion biology, molecular evolution, residual or disseminated-cell biology, and hepatic adaptation.

### 5.4. Treatment Implications and Limits

Stemness-associated markers may help shape future trial design, but they should not be used alone to select treatment at present. Treatment sequencing in metastatic breast cancer is still based on subtype, prior therapy, receptor status, actionable genomic alterations, disease burden, symptoms, organ function, patient preference, and drug access [4,5,6,7,8,52,57,58]. For BCLM, clinicians must also consider hepatic reserve, biliary involvement, liver-dominant progression, extrahepatic disease, and whether local treatment is feasible.

The near-term value of stemness-associated assessment is mainly translational. Persistent CTC clusters, stemness-enriched CTC phenotypes, or Claudin-2-associated liver-tropic signals after resection, ablation, or SBRT may identify biological features worthy of prospective evaluation, but they should not currently determine surveillance intensity or treatment selection. We do not suggest using these markers to intensify CDK4/6 inhibitors, antibody-drug conjugates, immunotherapy, or other systemic treatments outside validated indications. CD44-, ALDH-, Notch-, Wnt/beta-catenin-, Hedgehog-, and related stemness-targeted approaches remain investigational. They are relevant to residual, plastic, stress-tolerant, or niche-adapted BCLM states, but still require clinical validation [59,60].

## 6. Limitations and Future Directions

### 6.1. Limitations

Several gaps limit the clinical use of stemness-associated markers in BCLM. Many stemness studies come from primary breast tumors, cell lines, xenografts, or metastatic models that are not specific to the liver. By contrast, BCLM studies often focus on local treatment, survival, receptor changes, or general metastatic mechanisms [2,9,10,11,47,61]. Liquid biopsy studies also tend to group patients with different metastatic sites together. As a result, they rarely separate liver-limited disease, liver-dominant progression, or recurrence after liver-directed therapy. Few prospective studies have tested stemness markers, hepatic niche features, and clinical assessment tools in the same BCLM cohort.

Different types of evidence should not be treated as equivalent. Preclinical studies establish biological plausibility, such as TGF-beta1-induced expansion of CD44high/CD24− cells or Claudin-2-mediated hepatocyte interaction [19,21]. Retrospective cohorts support associations such as between Claudin-2 expression and early liver recurrence [22]. Clinical series, systematic reviews, and reconstructed individual-patient-data analyses provide context for local-therapy outcomes but remain vulnerable to patient-selection and other non-randomized biases [9,10,11]. Prospective BCLM-specific cohorts are needed before stemness-associated markers can be applied to clinical prediction or therapeutic selection.

### 6.2. Standardization Requirements for Assays and Endpoints

Translation requires standardization of assays, sampling procedures, and endpoints. Tissue-based studies should report antibody validation, scoring systems, thresholds, sampling sites, treatment status, and whether specimens are derived from the primary tumor, liver metastases, other metastatic sites, or post-treatment residual tissue. CTC assays should report enrichment method, EpCAM dependence, blood volume, cluster preservation, and marker panels. ctDNA assays should report sampling method, platform, detection limit, variant allele frequency rules, leukocyte filtering, and planned time points [48,49,50,51].

Clinical endpoints must also be BCLM-specific. OS and progression-free survival remain important, but studies should define time to liver-dominant progression, hepatic recurrence after local therapy, liver progression-free survival, deterioration of hepatic function, mixed response, receptor conversion, extrahepatic progression, and treatment resistance. A primary tumor marker used to estimate future risk of liver metastasis is not equivalent to a CTC marker measured during metastatic therapy or to a liver metastasis tissue marker assessed after local treatment. Clarification of sample source, disease phase, treatment exposure, and clinical question will improve reproducibility.

### 6.3. Future Studies in BCLM Cohorts

Future studies should be built around patients with confirmed or high-risk BCLM, rather than broad metastatic breast cancer cohorts. The most useful cohorts would collect paired primary tumors and liver metastases, serial blood samples for CTC and ctDNA testing, standardized liver imaging, and detailed clinical data. These data should include subtypes, receptor reassessment, treatment history, liver-directed therapy, liver function, extrahepatic disease, and survival or recurrence outcomes. With this design, researchers could test whether stemness-associated markers change during progression, increase after systemic or local therapy, or predict liver-dominant recurrence.

We also think future studies should address a practical clinical question. If a patient has stemness-associated CTC features but no definite radiologic liver progression, this result should not directly change treatment outside a trial. A safer approach is to include such patients in prospective studies after complete local control. These studies could test subtype-guided treatment optimization [52,57] or investigational anti-stemness strategies [59,60]. Imaging, CTC phenotype, ctDNA dynamics, and liver-specific endpoints should be prespecified. BCLM cohorts should also be stratified by subtype and disease pattern, such as HR-positive liver-dominant disease, HER2-positive visceral progression, and TNBC with early liver recurrence. Preclinical models remain useful, but they should be linked to clinical sampling time points and liver-specific outcomes [62].

### 6.4. Linking Local Therapy with Biological Monitoring

For BCLM, local control and biological monitoring should be considered together. Surgery, ablation, SBRT, TACE, SIRT, and other liver-directed treatments can control selected liver lesions or prolong disease-free intervals in some patients. Outcomes after liver-directed therapy remain influenced by disease extent, patient selection, hepatic reserve, and treatment modality [12,61,63,64,65,66,67,68,69]. Severe cumulative hepatic injury after repeated locoregional treatment has also been reported [70]. Conventional RFA series have reported high complete-necrosis rates in small selected lesions [66], whereas specialized SRFA cohorts have shown technical success rates close to 100% with local recurrence rates below 10% [12]. These settings are well suited for investigating residual-cell biology and hepatic recurrence.

Breast cancer stemness is not captured by any single marker. CD44+/CD24− populations have demonstrated tumor-initiating and invasive properties [16,71], while broader studies emphasize their heterogeneity and treatment context [17,28,59,60,72,73]. Importantly for BCLM, CD44s has also been shown experimentally to promote breast cancer metastasis to the liver [74]. More recent studies have linked HER2/HER3 signaling to trastuzumab resistance [75], Mcl-1 dependency to paclitaxel resistance [76], UCHL1 to TNBC stemness [77], and brown-adipocyte/IRX3 signaling to microenvironment-driven plasticity in HR-positive breast cancer [78]. These studies provide mechanistic context for treatment-modulated cellular states but do not establish BCLM-specific clinical biomarkers.

Liquid-biopsy studies likewise provide non-equivalent information. CTC transcriptomics illustrates phenotypic heterogeneity in metastatic breast cancer [79], while organ-specific CTC literature includes studies of bone metastasis [80] and CTC-xenograft modeling of a TNBC liver-metastasis signature [81]. Integrin profiles on exosomes and CTCs have also been associated with metastatic-site localization [82]. Broader reviews emphasize that ctDNA, CTCs, extracellular vesicles, and circulating RNA differ in biological content and standardization requirements [83,84,85,86]. Organotropism-focused work has separately examined miRNA cargo [87] and multi-organ CTC models [41], whereas epithelial collective mechanics [88], Afadin-associated adhesion [89], and the breast cancer secretome [46] provide additional context for invasion and niche remodeling. Future BCLM research should align preclinical models with well-annotated clinical samples, serial liquid biopsy, imaging, and liver-specific endpoints [62].

## 7. Conclusions

Breast cancer liver metastasis (BCLM) is shaped by both tumor-cell plasticity and adaptation to the hepatic microenvironment. Current evidence suggests that CD44/CD24 states, ALDH-related activity or isoform expression, EpCAM/CTC phenotypes, and CLDN2-associated liver tropism provide complementary information on dissemination, treatment tolerance, residual disease, and hepatic adaptation, but none is sufficiently validated for stand-alone clinical decision-making. These biomarkers should therefore be interpreted together with imaging, metastatic-site pathology, receptor reassessment, and clinically actionable genomic testing. At present, their main value is translational, particularly for defining biologically distinct residual or disseminated-cell states and developing longitudinal monitoring strategies. Future progress will require prospective BCLM-specific cohorts, standardized assays, paired primary and liver-metastatic specimens, serial liquid biopsy, and liver-specific endpoints. Such studies may determine whether stemness-associated biomarkers can ultimately refine risk stratification, post-treatment monitoring, and patient selection for future clinical trials.

## Figures and Tables

**Figure 1:**
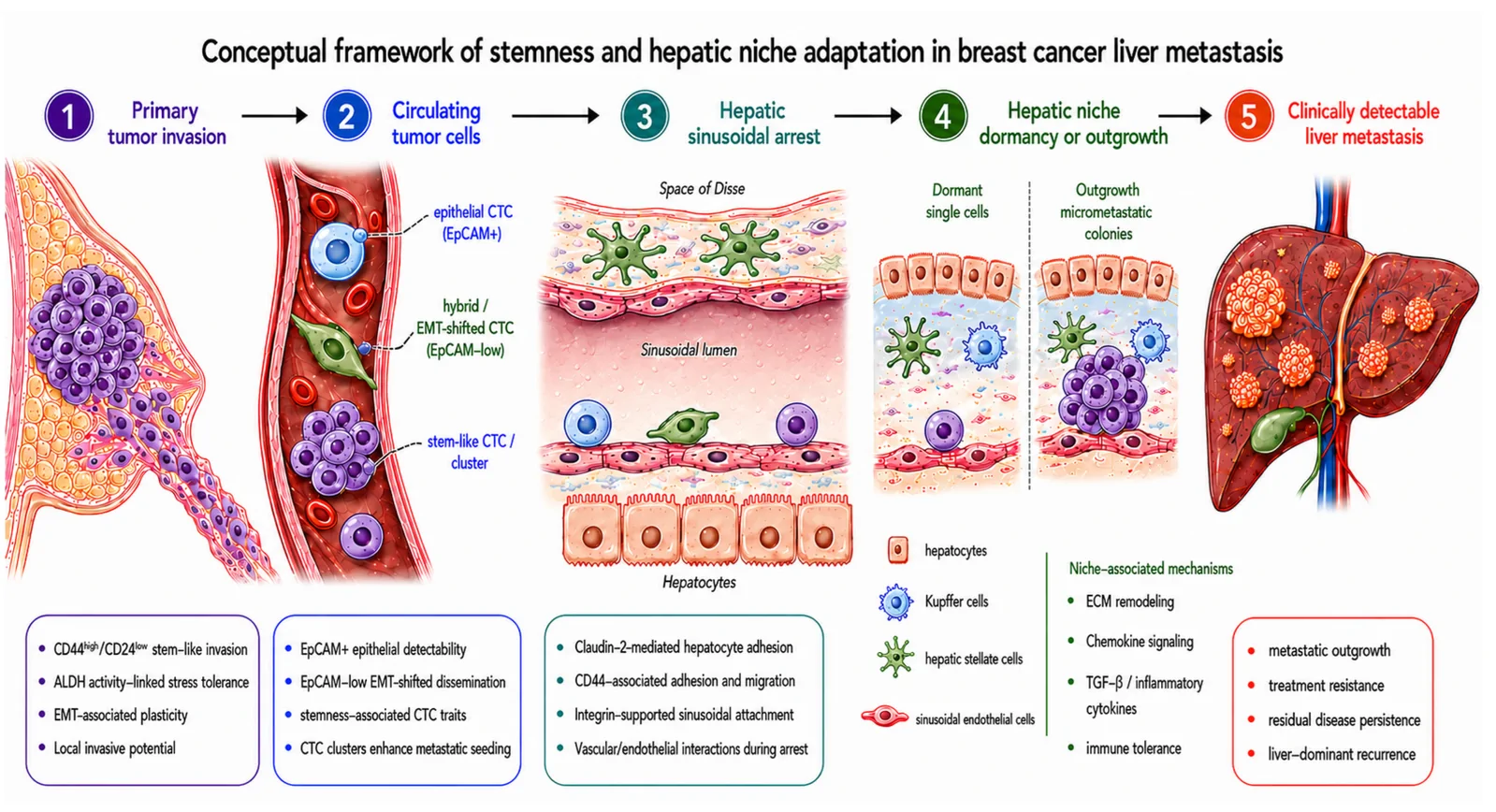
Conceptual framework of stemness and hepatic niche adaptation in breast cancer liver metastasis.

**Figure 2:**
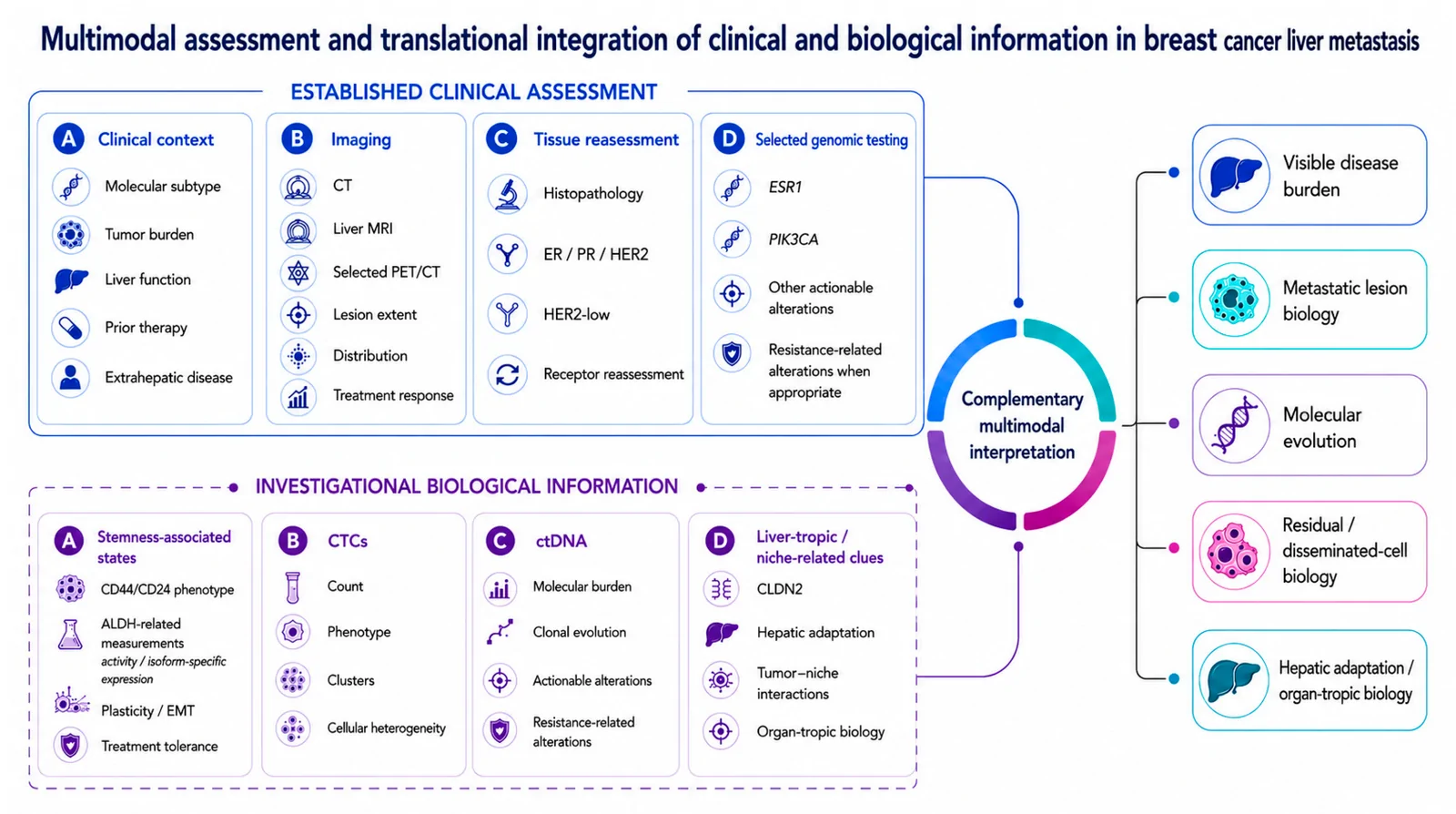
Multimodal assessment and translational integration of clinical and biological information in breast cancer liver metastasis. This schematic presents a descriptive framework for the complementary interpretation of clinical and biological information in breast cancer liver metastasis (BCLM). Established clinical assessment includes clinical context; imaging with CT, liver MRI, and selected PET/CT; tissue reassessment with histopathology and receptor evaluation; and selected genomic testing when clinically appropriate. Investigational biological information includes stemness-associated states, circulating tumor cell (CTC) features, circulating tumor DNA (ctDNA) dynamics, and liver-tropic or niche-related clues such as CLDN2. The dashed purple outline denotes the investigational status of these biological information layers. These non-equivalent but complementary modalities converge into multimodal interpretation of visible disease burden, metastatic lesion biology, molecular evolution, residual or disseminated-cell biology, and hepatic adaptation or organ-tropic biology. The investigational biomarkers shown have not been prospectively validated for BCLM-specific clinical decision-making and require further BCLM-specific validation before clinical integration. This framework does not define biomarker thresholds, individual patient risk categories, surveillance intervals, or treatment recommendations.

**Table 1:** Core stemness-associated and liver-tropic biomarkers for interpreting breast cancer liver metastasis.

Marker	Biology	Assay	Interpretation	Limitation	Source
CD44/CD24	Surface stemness and plasticity phenotype	Tissue IHC or flow cytometry; CTC or residual-cell phenotyping	Invasion, EMT plasticity, adhesion and residual-cell tolerance; not liver-specific	Variable cutoffs and subtype dependence; not sufficient for independent BCLM stratification	[15,18,19,27]
ALDEFLUOR-defined ALDH activity	Functional aldehyde-metabolizing activity associated with stem-like states	ALDEFLUOR flow cytometry with DEAB control	Enrichment of cells with tumor-initiating, stress-adaptive, or treatment-tolerant properties	Not isoform-specific; multiple ALDH isoforms can contribute to the signal; no validated BCLM-specific threshold	[28,29,30]
ALDH1A1 expression	Isoform-specific aldehyde metabolism and detoxification	ALDH1A1-specific IHC, protein assay, or transcript measurement	Isoform-specific biological/prognostic information	Must not be used as a surrogate for total ALDEFLUOR activity; associations depend on assay, subtype, and cohort	[29,31]
ALDH1A3 expression	Isoform-specific ALDH1A3 expression related to aldehyde metabolism	ALDH1A3-specific IHC, protein assay, or transcript measurement	Important contributor to ALDEFLUOR-positive phenotype in breast cancer; associated with aggressive/metastatic features in breast cancer studies	Not equivalent to total ALDH activity; not validated as a BCLM-specific prognostic marker	[29,30]
EpCAM/CTCs	Epithelial detectability and dissemination window	Peripheral blood CTC assays; EpCAM-based enrichment or alternative CTC platforms	Dynamic cellular monitoring of dissemination, phenotype, CTC clusters and partial EMT states	Rare events; EpCAM-low or EMT-shifted CTCs may be underestimated	[14,32,33,34,35]
Claudin-2	Liver-proximal adhesion and organotropism clue	Tissue IHC or transcript analysis; liver-tropic models; hepatocyte-adhesion assays	Tumor cell–hepatocyte interaction and hepatic colonization; more liver-related than generic stemness	No routine clinical cutoff; prospective BCLM validation limited	[20,21,38,39]

ALDH, aldehyde dehydrogenase; ALDH1A1, aldehyde dehydrogenase 1 family member A1; ALDH1A3, aldehyde dehydrogenase 1 family member A3; BCLM, breast cancer liver metastasis; CTC, circulating tumor cell; DEAB, *N*,*N*-diethylaminobenzaldehyde; EMT, epithelial–mesenchymal transition; EpCAM, epithelial cell adhesion molecule; IHC, immunohistochemistry.

## Data Availability

No new data were created or analyzed in this study. Data sharing is not applicable to this article.

## Abbreviations

The following abbreviations are used in this manuscript:

ADC	antibody-drug conjugate
ALDH	aldehyde dehydrogenase
ALDH1A1	aldehyde dehydrogenase 1 family member A1
ALDH1A3	aldehyde dehydrogenase 1 family member A3
ASCO	American Society of Clinical Oncology
BCLM	breast cancer liver metastasis
CDK4/6	cyclin-dependent kinase 4/6
cfDNA	cell-free DNA
CLDN2	claudin-2
CSCO	Chinese Society of Clinical Oncology
CT	computed tomography
CTC	circulating tumor cell
ctDNA	circulating tumor DNA
DEAB	*N*,*N*-diethylaminobenzaldehyde
EMT	epithelial–mesenchymal transition
EpCAM	epithelial cell adhesion molecule
ER	estrogen receptor
ESMO	European Society for Medical Oncology
ESR1	estrogen receptor 1
HER2	human epidermal growth factor receptor 2
HR	hormone receptor
IL-10	interleukin-10
JAK-STAT	Janus kinase-signal transducer and activator of transcription
MASLD	metabolic dysfunction-associated steatotic liver disease
MRI	magnetic resonance imaging
OS	overall survival
PET/CT	positron emission tomography/computed tomography
PI3K	phosphoinositide 3-kinase
PIK3CA	phosphatidylinositol-4,5-bisphosphate 3-kinase catalytic subunit alpha
PPARG	peroxisome proliferator-activated receptor gamma
PR	progesterone receptor
RFA	radiofrequency ablation
RNA	ribonucleic acid
SBRT	stereotactic body radiotherapy
SEER	Surveillance, Epidemiology, and End Results
SIRT	selective internal radiation therapy
SRFA	stereotactic radiofrequency ablation
TACE	transarterial chemoembolization
TGF-beta1	transforming growth factor beta 1
TNBC	triple-negative breast cancer
